# Annexin A2-mediated cancer progression and therapeutic resistance in nasopharyngeal carcinoma

**DOI:** 10.1186/s12929-018-0430-8

**Published:** 2018-03-29

**Authors:** Chang-Yu Chen, Yung-Song Lin, Chien-Ho Chen, Yin-Ju Chen

**Affiliations:** 1 School of Medical Laboratory Science and Biotechnology, College of Medical Science and Technology, Taipei Medical University, 250 Wu-Xing Street, Taipei, 11031 Taiwan; 2 Department of Molecular Preventive Medicine, Graduate School of Medicine, The University of Tokyo, Tokyo, Japan; 3 Department of Otolaryngology, School of Medicine, College of Medicine, Taipei Medical University, Taipei, Taiwan; 4 Department of Otolaryngology, Chi Mei Medical Center, Tainan, Taiwan; 5 Department of Radiation Oncology, Taipei Medical University Hospital, Taipei, Taiwan; 6 Graduate Institute of Biomedical Materials and Tissue Engineering, College of Biomedical Engineering, Taipei Medical University, Taipei, Taiwan; 7 International Ph.D. Program in Biomedical Engineering, College of Biomedical Engineering, Taipei Medical University, Taipei, Taiwan; 8 School of Biomedical Engineering, College of Biomedical Engineering, Taipei Medical University, Taipei, Taiwan

**Keywords:** Annexin A2 (ANXA2), Nasopharyngeal carcinoma (NPC), Cancer progression, Therapeutic resistance

## Abstract

Nasopharyngeal carcinoma (NPC) is a head and neck cancer with poor clinical outcomes and insufficient treatments in Southeast Asian populations. Although concurrent chemoradiotherapy has improved recovery rates of patients, poor overall survival and low efficacy are still critical problems. To improve the therapeutic efficacy, we focused on a tumor-associated protein called Annexin A2 (ANXA2). This review summarizes the mechanisms by which ANXA2 promotes cancer progression (e.g., proliferation, migration, the epithelial-mesenchymal transition, invasion, and cancer stem cell formation) and therapeutic resistance (e.g., radiotherapy, chemotherapy, and immunotherapy). These mechanisms gave us a deeper understanding of the molecular aspects of cancer progression, and further provided us with a great opportunity to overcome therapeutic resistance of NPC and other cancers with high ANXA2 expression by developing this prospective ANXA2-targeted therapy.

## Background

Nasopharyngeal carcinoma (NPC) is a low-frequency disease in western countries; however, it is a high-risk head and neck cancer in Southeast Asia and China [1, 2]. In the clinic, concurrent chemoradiotherapy improved overall survival (OS, 94.5% at 5 years) in stage I/II patients, but the same treatment did not work effectively in stage III/IV patients (OS, 72.3% at 5 years). With monotherapy, stage III/IV patients who received radiotherapy alone had a relatively poor survival rate (an OS of 54.2% at 5 years) [3–6]. To improve the therapeutic efficacy, we tried to understand the mechanism of therapeutic resistance. A meta-analysis of 2321 cancer patients with several cancer types including head and neck cancers (esophageal, sinonasal, and oral), showed that a high expression level of Annexin A2 (ANXA2) was related to poor overall survival and disease-free survival [7]. Consistent results of high expression levels of ANXA2 being associated with advanced patients were also found for NPC [8].

ANXA2, as a tumor-associated protein, promotes cancer progression including proliferation, invasion, and metastasis in various cancer types (NPC, ovarian cancer, gliomas, hepatomas, pancreatic cancer, and breast cancer) [8–13]. In addition to cancer progression, ANX family proteins (ANXA1 and ANXA2) suppress the efficacy of both chemotherapy and radiotherapy [8, 14, 15]. In 2015, we further uncovered the interaction of ANXA2 with dendritic cell (DC)-specific intracellular adhesion molecule (ICAM)-3 grabbing non-integrin (DC-SIGN, CD209), which resulted in immunosuppression. This suppression might influence outcomes of anticancer therapies [16].

In recent years, increased attention has focused on ANXA2 and its role in regulating cancer development [17–19]. In this review, cellular and molecular mechanisms of ANXA2-mediated cancer progression and therapeutic resistance are addressed in the first two sections. Then, we further discuss the prospective effect of ANXA2-targeted therapy in the final section.

## Cancer progression

### Aberrant expression in cancers

Over the period of 2014~ 2017, researchers pointed out that high expression of ANXA2 in biopsies of epithelial ovarian cancer (56.42%) [20], urothelial carcinoma (53.02%) [21], hepatocellular carcinoma (HCC; 73.81%) [22], NPC (33%) [8], and serous ovarian cancer (57.79%) [23] was associated with poor OS (Table 1). Weihua Qiu and his colleagues performed a meta-analysis of 2321 cancer patients to confirm that high expression of ANXA2 was correlated with both OS (hazard ratio [HR] 1.56; *p* <  0.001) and disease-free survival (HR 1.47; *p* <  0.001) [7]. High ANXA2 expression was also related to a high risk of metastases (*n* = 48, NPC) and recurrence (*n* = 93, endometrioid endometrial carcinoma) [8, 24]. On the other hand, ANXA2 serves as a diagnostic factor for screening cancers. In peripheral blood, the ANXA2 serum level has been evaluated in patients with HCC (median, 69.6 ng/ml) [25], early-stage HCC (median, 150 ng/ml) [26], gastric cancer (median, 211.0 ng/ml) [27], lung cancer [28], and oral squamous cell carcinoma (median, 27.1 ng/ml) [29] (Table 2). However, serum levels of ANXA2 in NPC patients have not yet been reported.

**Table 1:** High expression of Annexin-A2 (ANXA2) in biopsies as an indicator of the survival rate

Tumor type	*N*	High ANXA2 expression (%)	*p* value	Results	Reference
Epithelial ovarian cancer	119	65/119 (54.62%)	< 0.001	Poor survival rate	[20]
Nasopharyngeal carcinoma	48	32/48 (66.67%)	0.025	Poor survival rate	[8]
Urothelial carcinoma	232	123/232 (53.02%)	0.012	Poor survival rate	[21]
Hepatocellular carcinoma	84	62/84 (73.81%)	0.005	Poor survival rate	[22]
Serous ovarian cancer	109	63/109 (57.79%)	0.044	Poor survival rate	[23]

Method: immunohistochemistry; *N*, total number of patients

**Table 2:** High levels of Annexin-A2 (ANXA2) in serum as a potential biomarker for cancers

Tumor type	*N*	*p* value	Results	Reference
Hepatocellular carcinoma	70	< 0.001	Patients (*n* = 50, median, 69.6 ng/ml) Healthy individuals (*n* = 20, median, 9.5 ng/ml)	[25]
Hepatocellular carcinoma (early stage)	70	< 0.01	Patients (*n* = 50, median, 130 ng/ml) Healthy individuals (*n* = 20, median, 17 ng/ml)	[26]
Gastric cancer	93	< 0.001	Patients (*n* = 63, median, 211.0 ng/ml) Healthy individuals (*n* = 30, median, 120.5 ng/ml)	[27]
Lung cancer	85	< 0.01	Patients (*n* = 42) Healthy individuals (*n* = 43)	[28]
Oral squamous cell carcinoma	284	< 0.01	Patients (*n* = 126, median, 27.1 ng/ml) Healthy individuals (*n* = 158, median, 15 ng/ml)	[29]

Method: enzyme-linked immunosorbent assay; *N*, total number of patients

Additionally, circulating tumor cell (CTC) detection in peripheral blood has become a non-invasive way to predict the risk of developing metastasis in cancer patients. In 2015, Pomila Singh and her colleagues further used cancer stem cell (CSC) markers (e.g., doublecortin-like kinase 1 (DCLK1) and leucine-rich repeat-containing G-protein coupled receptor 5 (LGR5)) with epithelial cell markers (CD44 and ANXA2) to detect circulating CSCs in patients with malignant colon adenocarcinomas. CTC detection is an assay for on-going metastasis or relapse, while circulating CSC detection is a novel diagnosis during the initiation of metastasis. Detection of circulating CSCs provides a chance to diagnose metastasis and prevent it at earlier time points [30]. Due to broad approaches of biopsies, and serum and circulating CSC detection, ANXA2 might be a short-term clinical indicator for cancer patients.

### Proliferation

**Fig. 1:**
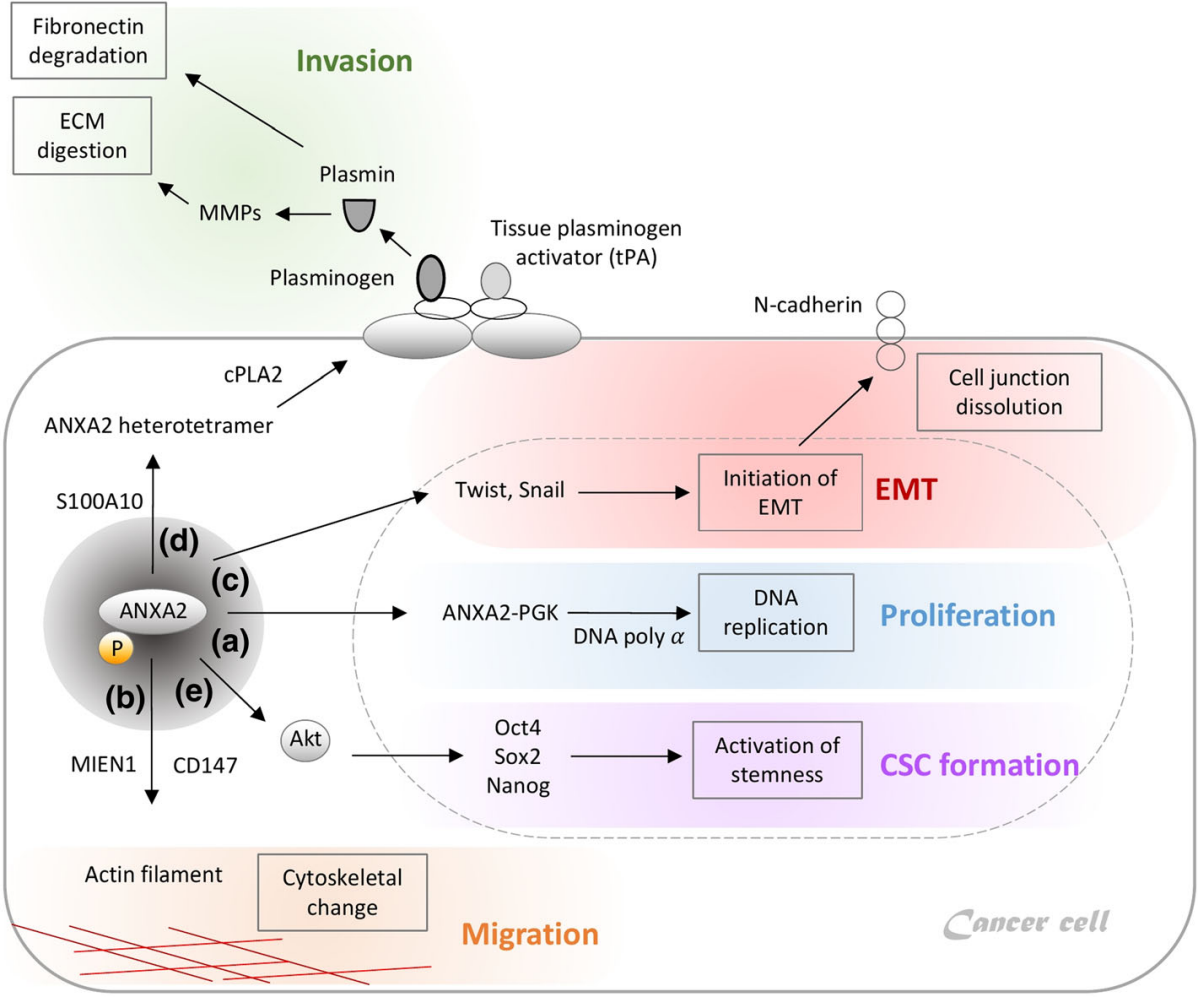
Annexin A2 (ANXA2) in cancer progression. **a** The ANXA2-3-phosphoglycerate kinase (ANXA2-PGK) complex serves as a primer recognition protein to initiate DNA replication with the support from DNA polymerase alpha, which contributes to cell proliferation. **b** MIEN1 phosphorylates ANXA2 and supports ANXA2’s binding to actin filaments to modulate cytoskeletal change, thus resulting in cell migration. **c** ANXA2 initiates the endothelial-mesenchymal transition (EMT) via the Twist/Snail pathway. After initiation of the EMT, cells changed to a mesenchymal-like morphology, and cell junctions dissolved. **d** The ANXA2 heterotetramer complex links to the plasminogen and tissue plasminogen activator (tPA). After plasminogen is cleaved into plasmin, plasmin activates pro-matrix metalloproteases (MMPs) to become MMPs. MMPs digest the extracellular matrix and fibronectin, thus resulting acceleration of invasion. **e** ANXA2 increases stemness-related transcription factors (Oct4, Sox2, and Nanog) through the Akt signaling pathway, which activates cancer stem cell formation

There are two types of ANXA2 in cells. The ANXA2 monomer exists in cell cytoplasm and nuclei, while the ANXA2 heterotetramer (consisting of two ANXA2 and two S100A10 molecules) exists on cell membranes. In nuclei, the ANXA2 monomer combines with 3-phosphoglycerate kinase (PGK) to become a complex. This complex performs the same function as primer-recognition proteins to stimulate DNA polymerase alpha, which contributes to the initiation of DNA replication in the early S phase in cervical cancer cells (Fig. 1a) [31]. In addition to promoting DNA replication, nuclear ANXA2 disrupts coilin causing it to abnormally localize to centromeres, thereby precipitating chromosome instability (CIN) which was demonstrated in human colorectal cancer (CRC) cells (Caco-2, HCT116, SW480, DLD-1, and RKO). Chromosome instability was reported to accelerate tumor growth and contribute to cellular resistance to chemotherapy [32–34].

### Migration

Phosphorylation of the tyrosine 23 (Tyr23) residue on ANXA2 is a key regulator controlling cell mobility. The migration and invasion enhancer 1 (MIEN1) interacts with ANXA2 to enhance Tyr23 phosphorylation on ANXA2. Phosphorylated ANXA2 binds to actin filaments on cell membranes, and modulates cell scattering and cytoskeletal changes via actin remodeling in human breast cancer cells (SK-BR-3 and BT-474). A phosphorylation deficiency of Tyr23 and Tyr50 causes cells to lose the ability to migrate in in vitro wound healing assays [35–38]. However, a different report showed that the extracellular matrix metalloproteinase (MMP) inducer (also known as CD147) prohibits Tyr23 phosphorylation on ANXA2, and promotes cell migration via suppressing ANXA2-DOCK3-β-catenin-WAVE2 step-by-step signaling in human hepatoma cells (SMMC-7721, HuH-7, and HepG2) [39]. It seems that either the promotion of Tyr23 phosphorylation by MIEN1 or the inhibition of Tyr23 phosphorylation by CD147 eventually contributes to cell migration (Fig. 1b). The precise role of Tyr23 phosphorylation in ANXA2’s actions requires further investigation.

### Endothelial-mesenchymal transition (EMT)

The EMT is a normal morphogenic process during embryonic development and tissue restructuring; however, the EMT is also the initial step in metastasis [7]. Twist and Snail are two critical transcription factors that promote the EMT in cancers. In EMT initiation, Twist and Snail decrease epithelial proteins (i.e., E-cadherin) and increase mesenchymal proteins (i.e., N-cadherin, fibronectin, β-catenin, and vimentin). Cancer cells without adherent junctions (i.e., E-cadherin) can gain an advantage to metastasize from one organ to a different indirectly connected one [40]. Phosphorylation of the Tyr23 residue on ANXA2 was reported to be an initiator of the EMT (mediated by Rho or induced by transforming growth factor (TGF)-β) [12, 37, 41]. In ANXA2-knockdown NPC cells, expression levels of Twist and Snail decreased. ANXA2-knockdown NPC cells retained the endothelial-like phenotype rather than changing to a mesenchymal-like one. This result indicated that ANXA2 is a critical factor in initiation of the EMT via the Twist/Snail signal pathway (Fig. 1c) [8].

### Invasion and metastasis

ANXA2 promotes the invasion and metastasis of different cancers (ovarian cancer, HCC, gliomas, pancreatic cancer, renal cell carcinoma, lung cancer, breast cancer, and NPC) [8–12, 42–44]. The mechanism of the association of the ANXA2/S100A10 heterotetramer with extracellular matrix (ECM) digestion was described in previous studies [45–47]. Here, we update recent advances and more precisely summarize the mechanism. First, cytoplasmic phospholipase A2 (cPLA2) binds to the ANXA2/S100A10 heterotetramer in the cytoplasm to assist ANXA2 in translocating to cell membranes. On cell membranes, S100A10 links to the plasminogen and tissue plasminogen activator (tPA). S100A10 is required for the plasminogen-dependent oxidation of the ANXA2/S100A10 heterotetramer. Oxidation facilitates the catalytic cleavage of plasminogen to plasmin between arginine (Arg)561 and valine (Val)562 by the active tPA [48]. Plasmin cleaves the tissue inhibitor of metalloproteinases on pro-matrix metalloproteases (MMPs), and then the pro-MMPs become the active forms of MMPs. MMPs (e.g., MMP-2 and MMP-9) degrade fibronectin and the ECM, thus resulting in acceleration of invasion and metastasis (Fig. 1d) [49–52].

### CSC formation

CSCs are cancer cells with stem-like properties to initiate self-renewal and differentiation. CSCs promote invasion, metastasis, suppress chemotoxicity and radiotoxicity [53–55]. In the previous section on aberrant expression in cancers, we described how ANXA2 can be a novel marker to detect circulating CSCs in the clinic; however, the role of ANXA2 in CSCs is not yet clearly understood. As we know, the Akt protein upregulates stemness-related transcription factors (Oct4, Sox2, and Nanog) [56–58] and is involved in CSC maintenance in different cancer types (gliomas, esophageal carcinoma, and non-small cell lung cancer) [59–61]. To further determine the mechanism between Akt and ANXA2, we established ANXA2-knockdown NPC cells. After ANXA2 silencing, the amount of active Akt decreased, thus indirectly suppressing protein levels of stemness-related transcription factors (Oct4, Sox2, and Nanog). Without sufficient stemness-related transcription factors, ANXA2-knockdown NPC cells are likely to lose their CSC properties (Fig. 1e) [8].

## Therapeutic resistance

### Correlations between the ANX family and therapeutic resistance

Radiotherapy and concurrent chemoradiotherapy are general strategies for NPC; however, we found resistance to both radiotoxicity and chemotoxicity in NPC cells with high ANXA2 expression. In addition to ANXA2, high expressions of other Annexin proteins were also reported to be associated with therapeutic resistance in different cancers, such as ANXA1 against chemo−/radiotherapy in the CNE2 NPC cell line [14, 15], ANXA2 against multiple drugs in NPC, gastric cancer, breast cancer, and pancreatic cancer [8, 62–64], ANXA3 against chemotoxicity in HCC patients (*n* = 34) [65], and ANXA4 against chemotherapeutic drugs in human ovarian (OVTOKO and OVISE), lung (H460) and colorectal (HCT15) cancer cells [66, 67]. Conversely, low ANXA11 expression is related to ovarian cancer with cisplatin resistance [68]. A correlation between therapeutic resistance and the Annexin protein family was found; nevertheless, the mechanism is still unclear. To improve ANAX2’s therapeutic efficacy against NPC, it is necessary to understand the mechanism of how it enables tumor cells to suppress radiotoxicity or chemotoxicity.

### Resistance to chemotherapy

**Fig. 2:**
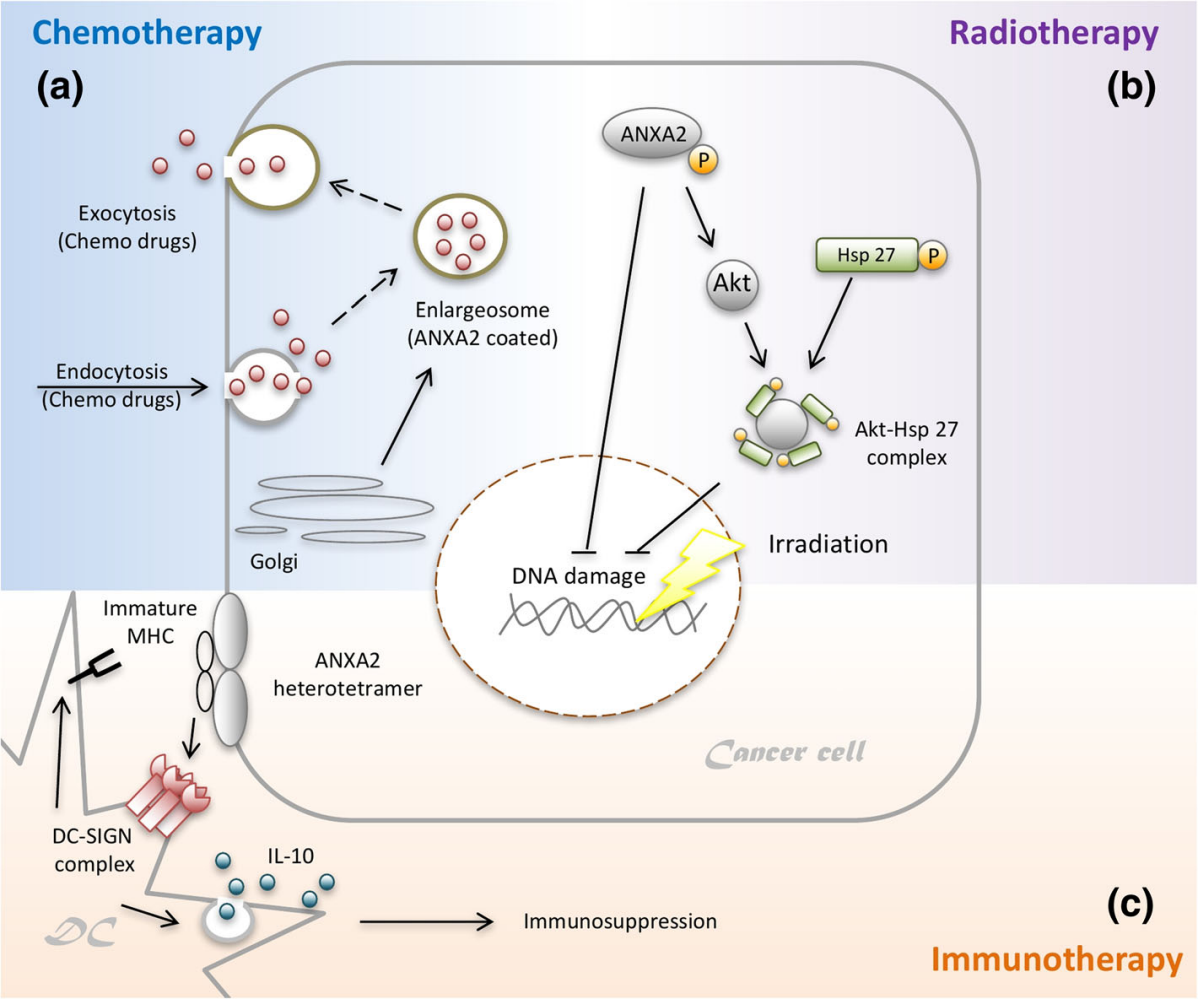
Annexin A2 (ANXA2) in therapeutic resistance**. a** ANXA2-coated enlargeosomes widely exist in various cell types to regulate Ca^2+^-dependent cell exocytosis. Enlargeosomes exocytose chemotherapeutic drugs to prevent their chemotoxic accumulation inside tumor cells, thus resulting in chemotherapeutic resistance. **b** The phosphorylated ANXA2 protein is imported into nuclei to protect against DNA damage by irradiation. ANXA2 also mediates the Akt protein to form the Akt-heat shock protein 27 (Akt-HSP27) complex, which ameliorates radiotoxicity-induced DNA damage and apoptosis. **c** When tumor-infiltrating dendritic cells (DCs) are attached to nasopharyngeal carcinoma (NPC) cells, the interaction between DC-SIGN and ANXA2 causes DCs to lose mature major histocompatibility complex (MHC), and release high levels of the immunosuppressive cytokine interleukin (IL)-10. IL-10 causes consecutive immunosuppressive responses including DC immaturity, inhibition of IL-12 synthetic, CD8^+^ T cell dysfunction, and regulatory T cell expansion

Human NPC cells with high ANXA2 expression can potentially resist different chemotherapeutic drugs (cisplatin, 5-fluorouracil, docetaxel, and vincristine). ANXA2-coated enlargeosomes widely exist in various cell types to regulate Ca^2+^-dependent cell exocytosis. Knockdown of ANXA2 in tumor cells might decrease the number of and limit the function of enlargeosomes. Dysfunctional enlargeosomes allow chemotherapeutic drugs to condense in the cytoplasm, thus resulting in tumor death (Fig. 2a) [8, 69–71]. Furthermore, intracellular ANXA2 binds the p50 subunit of nuclear factor (NF)-κB to become the ANXA2-p50 complex when pancreatic cancer cells (MIA-PaCa-2) are exposed to genotoxic agents (such as gemcitabine). This complex can be translocated to nuclei to activate the NF-κB signaling pathway. Activated NF-κB has multiple roles in cancer progression through modulating cell apoptosis and drug resistance. On the other hand, Qing-Yong Ma and his colleagues discovered that the phosphatidylinositol-3-kinase (PI3K)/Akt/NF-κB signaling pathway is activated by the interaction of ANXA2 and tenascin-C on the surface of pancreatic cancer cells (AsPC-1, PANC-1, and MIA-PaCa-2), which suppresses gemcitabine-induced cytotoxicity [72–74].

### Resistance to radiotherapy

In 2012, David M. Waisman and his colleagues indicated that the ANXA2 protein was imported into nuclei to protect against DNA damage caused by irradiation in human breast and lung cancer cells. ANXA2 is mainly localized in the cytoplasm and plasma membranes, and only a small amount of it is imported into nuclei. ANXA2 contains a leucine-rich nuclear export signal (NES) in its N-terminal domain. In the NES motif, lysine (Lys)10 and Lys12 are two critical residues which prevent ANXA2 from accumulating in nuclei. However, inducers such as gamma-radiation, ultraviolet radiation, etoposide, chromium VI, hydrogen peroxide (H_2_O_2_), and reactive oxygen species (ROS) may induce ANXA2 phosphorylation, which allows it to be translocated into nuclei [75, 76]. Phosphorylation regulates ANXA2’s export from and import into nuclei. Phosphorylation of the serine (Ser)11 and Ser25 residues allows ANXA2 to be exported from nuclei; however, phosphorylation at the Tyr23 residue contrastingly allows it to be imported into nuclei (Fig. 2b) [77, 78]. Different phosphorylation sites on ANXA2 can cause totally opposite results. In 2015, we investigated the downstream signaling pathway of ANXA2 in NPC cells. ANXA2 is involved in the Akt pathway and indirectly increases the number of Akt proteins [8]. When exposed to irradiation, the Akt protein binds to heat shock protein 27 (HSP27) to become the Akt-HSP27 complex, which ameliorates radiotoxicity-induced DNA damage and apoptosis (Fig. 2b) [79].

### Resistance to immunotherapy

A range of novel immunotherapies for cancers are under evaluation. Strategies for NPC were reported and are divided into two streams. First, Epstein-Barr virus (EBV)-specific cytotoxic T lymphocytes (CTLs) have become an effective adoptive cell therapy (ACT). The EBV latent membrane protein 1 (LMP1) is expressed on EBV-infected carcinoma cells. In 2014, Dennis J Moss and his colleagues transferred autologous CTLs that specifically targeted LMP1-expressing carcinoma cells into patients with recurrent NPC. After an injection, most of the pulmonary lesions disappeared, but the primary tumor did not regress [80]. To improve the disadvantage of targeting LMP1 alone, a new adenoviral vector was designed. This new adenoviral vector inserted EBV LMPs and EBV nuclear antigen-1 (EBNA1), which expanded specific CTLs against LMP- and/or EBNA1-expressing NPCs [80–83]. Second, DC-based immunotherapy is another option. DCs present tumor antigens to naïve CD8^+^ T cells in draining lymph nodes, and then naïve CD8^+^ T cells turn into tumor-specific CTLs [84–88]. However, NPC cells could give rise to suppressive responses after cell-cell interactions with DCs, thus resulting in immune escape [16, 89–91]. DCs can sense tumor-derived factors through receptors in both extracellular and intracellular milieus. Receptors include intracellular helicases, surface/intracellular toll-like receptors (TLRs), and surface C-type lectin receptors (CLRs). CLRs capture pathogen-associated molecular patterns (PAMPs) and endogenous ligands. DC-SIGN, a kind of C-type lectin, is composed of a carbohydrate recognition domain (CRD), a neck region with seven repeats, and a transmembrane region with a cytoplasmic tail [92–96]. DC-SIGN recognizes N-acetylglucosamine, mannose, fucose, and non-sialylated Lewis structures by CRD [95]. Mannosylated lipoarabinomannan (ManLAM) induces DC-SIGN downstream transcription factors (such as Ras, Raf-1, and NF-κB) to increase interleukin (IL)-10 promoter activity [97, 98]. After DC-SIGN is ligated by mannose- or fucose-containing oligosaccharides, it indirectly increases IL-10 production via the Th2 pathway in DCs. Normally, IL-10 suppresses prolonged and exaggerated immune responses [99, 100]. However, when DCs attach to NPC cells with high ANXA2 expression, the strong interaction between DC-SIGN and ANXA2 causes DCs to release extremely high levels of IL-10 (Fig. 2c). Once IL-10 spreads into the tumor environment, it causes consecutive immunosuppressive responses including DC immaturity (losing the major histocompatibility complex and the co-stimulatory molecules), the IL-12 synthetic inhibition, CD8^+^ T cell dysfunction, and regulatory T cell expansion [101–107].

## Prospective ANXA2-targeted therapy

From 2013, different research teams began to develop ANXA2-targeted therapy. C Ricciardelli’s team used an anti-ANXA2 antibody to reduce both tumor growth and metastasis in an ovarian cancer mice model (SK-OV3) [9]. One year later, Mandip Singh and his colleagues inserted short hairpin (sh)RNA targeting ANXA2 (shANXA2) into a cationic ligand-guide (CLG, a liposomal carrier) to construct a CLG-ANXA2 compound. The CLG-ANXA2 was designed to recognize cancer cells and CSCs in a lung cancer mouse model (H1650). After CLG-ANXA2 was taken up by tumor cells, shANXA2 prohibited ANXA2 messenger (m)RNA expression and decreased its protein level. The CLG-shANXA2 group showed inhibited tumor growth (reduced 72%~ 75% relative to the control, *p* <  0.001) [108]. To verify the above results, we established ANXA2-knockdown NPC cell lines by shRNA. Proliferation, migration, adhesion, and CSC formation were indeed reduced in ANXA2-knockdown NPC cells. Moreover, ANXA2-knockdown NPC cells lost the ability to suppress chemotoxicity, radiotoxicity, and immune responses [8, 16]. Targeting ANXA2 raises the possibility of being able to overcome the low therapeutic efficacy of cancers with high ANXA2 expression.

In addition to ANXA2, high expression of the epidermal growth factor receptor (EGFR) was previously discovered in NPC cells [109, 110]. In 2005, combined therapy with cetuximab (as the first chimeric anti-EGFR antibody) and carboplatin was used against NPC in 60 patients. After treatment, only 11.7% of patients had a response, and 48.3% had a stable disease rate [111]. It seems that treatment with cetuximab and carboplatin needs more consideration due to its low efficacy. In 2017, Liming Huang and his colleagues reported appealing results that silencing ANXA2 would reverse the EGF-induced EMT and inhibit cell migration in epidermoid cervical carcinoma cells (Ca-Ski, HeLa, and SiHa) [112]. ANXA2 helps the tyrosine-protein kinase transforming protein, Src (v-Src), mediate actin-cytoskeletal rearrangement which enhances proliferation, migration, and viability through the EFGR pathway [113]. The above findings gave us a new selection to combine anti-ANXA2 and anti-EGFR antibodies to fight against double-positive NPC (EGFR^+^/ANXA2^+^).

Nevertheless, ANXA2-deficient (ANXA2^−^/^−^) mice showed an increased risk of thrombosis and a decreased ability of neoangiogenesis [47]. Although ANXA2-targeted therapy suppresses cancers, it may produce side effects in patients. Thus, it is necessary to consider the expression titer of ANXA2, the dose level of ANXA2-targeted antibodies (or carrier with shANXA2), and the patient’s health condition before using ANXA2-targeted therapy.

## Conclusions

This review reveals the cancerous and suppressive mechanisms of ANXA2. First, we stepwise described the mechanisms of how ANXA2 promotes proliferation, migration, the EMT, metastasis, invasion, and CSC formation. On cell membranes, the ANXA2-S100A10 heterotetramer promotes activation of MMPs to increase the invasive ability. In cytoplasm, ANXA2, after being phosphorylated at Tyr23, binds to actin filaments to enhance migration. Inside cell nuclei, ANXA2 promotes both EMT- and CSC-related transcriptional factors (Snail, Twist, Oct4, Sox2, and Nanog). Second, we illustrated different therapeutic resistances by ANXA2. After phosphorylation, ANXA2 is translocated into nuclei and prevents DNA damage due to radiotoxicity. ANXA2-coated enlargeosomes exocytose chemotherapeutic drugs to decrease the level of chemotoxicity in cancer cells. Moreover, the molecular interaction of ANXA2 and DC-SIGN triggers immunosuppression, which results in tumor immune escape. The above findings gave us a deeper understanding of the molecular aspects of cancer progression, and provided a great opportunity to improve the therapeutic efficacy against NPC and other cancers with high ANXA2 expression. Although the ANXA2-targeted therapy has not been examined in clinical trials yet, it is expected to produce promising treatment outcomes.

## Abbreviations

ANXA2: Annexin A2
Arg: Arginine
CLG: Cationic ligand-guided
CLR: C-type lectin receptor
cPLA2: Cytoplasmic phospholipase A2
CRC: Colorectal cancer
CRD: Carbohydrate recognition domain
CSC: Cancer stem cell
CTC: Circulating tumor cell
DC: Dendritic cell
DC-SIGN: Dendritic cell-specific EBV Epstein-Barr virus
DFS: Disease-free survival
DLCK1: Doublecortin-like kinase 1
EBNA1: EBV nuclear antigen-1
ECM: Extracellular matrix
EGFR: Epidermal growth factor receptor
EMT: Endothelial-mesenchymal transition
H_2_O_2_: Hydrogen peroxide
HCC: Hepatocellular carcinoma
HR: Hazard ratio
HSP27: Heat shock protein 27
ICAM: Intracellular adhesion molecule
ICAM-3: Grabbing non-integrin
IL-10: Interleukin-10
LGR5: Leucine-rich repeat-containing G-protein coupled receptor 5
LMP: Latent membrane protein
Lys: Lysine
MIEN1: Migration and invasion enhancer 1
MMP: Matrix metalloproteases
NES: Nuclear export signal
NPC: Nasopharyngeal carcinoma
PAMP: Pathogen-associated molecular pattern
PGK: Phosphoglycerate kinase
PI3K: Phosphatidylinositol-3-kinase
ROS: Reactive oxygen species
Ser: Serine
shANXA2: Short hairpin RNA targeting Annexin A2
TGF: Transforming growth factor
tPA: Tissue plasminogen activator
Tyr: Tyrosine
Val: Valine
